# Social support in low-income women with Fibromyalgia Syndrome from a sub-urban and peri-urban areas of Tenerife (Canary Islands, Spain): a mixed method study

**DOI:** 10.1186/s41687-023-00661-0

**Published:** 2023-12-22

**Authors:** Sebastian Eustaquio Martín Pérez, Isidro Miguel Martín Pérez, Ania Álvarez Sánchez, Paula Acosta Pérez, Eliana Rodríguez Alayón

**Affiliations:** 1 Musculoskeletal Pain and Motor Control Research Group, Faculty of Health Sciences, Universidad Europea de Canarias, Santa Cruz de Tenerife, 38300 Spain; 2 Musculoskeletal Pain and Motor Control Research Group, Faculty of Sport Sciences, Universidad Europea de Madrid, Villaviciosa de Odón, Madrid, 28670 Spain; 3 Escuela de Doctorado y Estudios de Posgrado, Universidad de La Laguna, Santa Cruz de Tenerife, 38203 Spain; 4 Departamento de Farmacología y Medicina Física, Área de Radiología y Medicina Física, Facultad de Ciencias de la Salud, Universidad de La Laguna, Santa Cruz de Tenerife, 38200 Spain

**Keywords:** Fibromyalgia, Pain, Women, Low-incomes, Social support, Social networking

## Abstract

**Background:**

Women with Fibromyalgia Syndrome (FMS) can benefit form adequate social support to fight the consequences of their illness, but the extent to which this is available to those with low incomes who live in depressed areas of Tenerife (Canary Islands, Spain) is currently unknown. The purpose of this study was to explore social support in low-incomes women with FMS in sub-urban and peri-urban areas of Tenerife.

**Methodology:**

A sequential exploratory mixed method study was carried out from January 20, 2023, to June 10, 2023, at the Fibromyalgia and Chronic Fatigue Association of Tenerife (AFITEN) using non-probability convenience sampling. Social support was analyzed quantitatively through MOS-SSS survey and Duke-UNC-11 questionnaire, while qualitative data were obtained through semi-structured interviews to identify social support providers and analyze their satisfactions levels.

**Results:**

A total of 49 women, with a mean age of 57.80 years-old (SD = 13.25) were finally included in this study. MOSS-SSS and Duke-UNC-11 both indicated lower social support levels at 68.6 (SD =16.3) and 38.0 (SD = 9.74), respectively. The qualitative analysis revealed that partners and friends provided the most significant support with the highest satisfaction scores.

**Conclusions:**

The socioeconomic status of low-income women with FMS living in sub-urban and peri-urban areas of Tenerife (Canary Islands, Spain) influences on their social support, with the affective support and confidentiality being the most affected dimensions.

## Background

Fibromyalgia Syndrome (FMS) is a common disorder that tends to affect women more frequently than men, and is characterized by widespread chronic pain, cognitive and affective disturbances, and a persistent sensation of fatigue [1]. FMS is a good representative of diseases that cannot be attributed to a single biological cause such as tissue damage. Instead, it is influenced by a multitude of psychological factors included psychological emotion, thoughts, beliefs as well as social factors [2, 3]. These patients face an array of restrictions and difficulties that have a strong impact on their daily functioning, financial situations, and mental well-being. These obstacles are likely to have a negative effect on their ability to engage to society and their overall quality of life [4, 5]. After conducting a focus group study in study we determined that social support from one’s environment is crucial in accepting the disease. This is further supported by the fact that a patient’s environment plays a significant role in managing the disease, as cited in our study [6]. Social is defined as the resources that one perceive as available within their own social network [7]. The nature of social support is predominantly subjective, as it depends on the patient’s personal perception of emotional support, material aid and the provision of advice and information [8]. Bolwijn et al. (1996) [9] have suggested that examining the characteristics of familial and community support networks can provide valuable insights into individual social behavior. Although several research have proven the importance of social support in combating this specific disease, a number of these studies are restricted in their epistemological scope and methodology.

Our study on the social support perceived by women with FMS stands apart from other research in two significant ways. Firstly, we focus specifically on this particular population, which has not been extensively studied in terms of social support. Several studies have examined the prevalence of sexual dysfunction among women with FMS [10]. One of these studies focused specifically on Turkish women [11], while another was conducted with a sample of adolescents with the disease in Ontario, Canada [12]. Additionally, research has recently been conducted on men with FMS [13].

Not enough attention has been directed towards women from lower socioeconomic strata that suffer from FMS. The presence of poverty predominantly affects those who belong to lower social strata. The notion that poverty and illness are inextricably linked is a well-known phenomenon, resulting in a cycle of deprivation in which one factor is suitable to exacerbate the other [14]. Consequently, these circumstances serve as a barrier to accessing healthcare services in numerous ways. Patients with severe care needs, such as those who are affected with FMS, are often unable to access expensive treatments. In such situations, social support assumes a pivotal role in mitigating the cost of the disease. Despite previous attempts, the issue of social support amongst Spanish women with FMS from lower socioeconomic stratum remains ambiguous.

The second novelty is the application of a novel and reliable method. The studies published to date present important limitations in terms of their epistemological scope and methods. This need has been noted previously by Franks and Cronan (2004) [15] who affirmed that quantitative designs have restrictions when it comes to providing relevant data about the elements that impact the welfare of patients suffering from FMS. Rather than utilizing quantitative approaches, they argue that qualitative methods are better suited to identifying and understanding the operational components that compose relationships that support patients. These relationships are crucial in facilitating coping mechanisms and promoting social integration within the community.

From an epistemological perspective, social support has been studied by many theories, such as Gadamer’s philosophical hermeneutics. To study social support, interpretive or qualitative are the most commonly used paradigms [10].

Previously, a qualitative descriptive study has already been employed to describe the way individuals with FMS cope with the pain they feel while still managing to live. Specifically, they documented the use of non-pharmacological strategies or combinations of pharmacological and non-pharmacological treatments. Another method of research is the group interview approach [16]. In other cases, social support was studied through individual psychiatric semi-structured interviews that were made by a psychiatrist. [11] From them, information was gathered through semi-structured interviews and, in another instance, through depth interviews [18]. Authors sometimes combine different quantitative methods to examine social support. For example, Gündüz et al. (2018) [11] combined semi-structured interviews and structured interviews with questionnaires, while Bolwijn et al. (1996) [9] used a self-report questionnaire in their study. On the other hand, cross-sectional, retrospective and case-control studies are employed to assess the social network characteristics of patients with FMS and their perceived feelings of loneliness [9]. About mixed methods, they have been employed in multiple investigations and disciplines. Previous research have studied the concept of perceived social support in patients with mental illness using this approach. Mixed design study was also employed to investigate how social support and healthcare support affect the quality of life of individuals with FMS and Chronic Fatigue Syndrome [19]. Additionally, a study that employed an explanatory sequential mixed method was conducted by West et al. (2012) [20] to identify the strengths or resilience of families dealing with chronic pain. In 2018, Fernández-Peña* et al*. [21] used a mixed method to assess the effectiveness of the Personal Network Analysis as a means for investigating the social support associated with chronic pain. Another study employed a hybrid methodology to explore the way that women are affected by the disease as it progresses. This was done through in-depth interviews and the impact of the disease in terms of profile (SIP)[4]. All of these studies utilized a mixed method that employed two different approaches for analysis. In this method, the qualitative component is conducted using a constant comparison method, while the quantitative component is analyzed using a descriptive correlational method [19]. When studying the social support of people with FMS in Spain, the majority of the research is conducted within the quantitative paradigm.These works are usually observational and have a cross-sectional design that is composed of surveys or questionnaires, which only superficially explore the complex nature of this phenomenon [22, 23]. Furthermore, while some investigations have been conducted in both urban and suburban areas, none have specifically examined the association between socioeconomic status and social support. This implies that there is a significant bias in the other author´s findings. Additionally, the social support in island systems with partial industrialization in the outermost parts of Europe is currently unknown. Previous studies have not examined the specific way in which these structural and functional factors affect social support in low-income women with FMS. The purpose of this study is to explore social support in low-income women with FMS in sub-urban and peri-urban areas of Tenerife (Canary Islands, Spain). The first phase that quantifies the support received by women with FMS in a sample of sub-urban and peri-urban residents of Tenerife will consist in quantifying this support. The findings will be enhanced through a second phase that is qualitative that will involve the identification of the primary social support providers who provide emotional, physical and informational assistance as well as the degree to which the participants appreciate them.

## Methodology

### Study design

We used a mixed study based on a parallel and convergent sequential exploratory mixed method in order to answer our research question. Our aim through this design is, on the one hand, to decrease the presence of “*inappropriate uncertainty*” in order to enhance the trustworthiness of our conclusions and, in the other hand, to offer a deeper comprehension of complex phenomenon [24]. During the initial phase, the method involved the collecting, analyzing and comprehension of quantitative data. This was achieved through the utilization of scales to gather information from participants regarding their individual experiences with social support. The second stage involved conducting semi-structured interviews to identify those who provide support, analyze their level of satisfaction, and consequently, obtain an understanding of their experiences and perspectives.

.This study was conducted from January 20, 2023 to June 10, 2023 at Fibromyalgia and Chronic Fatigue Association of Tenerife (Canary Islands, Spain) and was approved by Ethics Committee 23/112-OS-X-TFM. Before participating in the study, each participant signed an informed consent (IC). By accepting the IC, individuals agree to be interviewed and have their testimonials recorded in audio. They also agreed that their data would be included in an anonymized database for research purposes.

### Study subjects

Non-probability convenience sampling was carried out to recruit low-income women with FMS in a sub-urban and peri-urban areas of ​​Tenerife (Spain). Upon receiving the signed IC, the researcher A.A.S. proceeded with a clinical interview to determine whether the candidate met the study’s inclusion criteria that were: (1) female, (2) over 18 years of age, (3) with medical diagnosis of FMS, (4) and being registered in the Fibromyalgia and Chronic Fatigue Association of Tenerife (AFITEN), (5) being categorized as low-income by a Social Worker, (6) living in the conurbation of Santa Cruz de Tenerife and San Cristóbal de La Laguna, municipalities located in the island of Tenerife (Canary Islands, Spain), (7) having not visual or hearing disability or neurological or psychiatric disorder that would interfere their ability to understand or speak Spanish, and (8) giving the consent to participate in the study.

### Collection of information and measuring instruments

After reviewing eligibility criteria and IC approval for participation in the study, P.A.P. collected variable data between March 2, 2023, and April 1, 2023. The information included demographic data such as gender, age, nationality, marital status, educational background, employment status, type of labor contract and place of residence.

### Study variables

Regarding quantitative approach, the Medical Outcome Study Social Support Survey (MOS-SSS) [25] was used. This survey, which consists of 9 items in its Spanish version, evaluates social support through various dimensions such as emotional and informational support, constructive social interactions, tangible aid as well as emotional assistance. The answer options are: 0 = “*never*”, 1 = “*rarely*”, 2 = “*sometimes*”, 3 = “*most of the time*” and 4 = “*all the time*”. 

The Duke-UNC Functional Social Support (Duke-UNC-11) questionnaire [26] was also used to measure additional emotional dimensions of social support such as confidentiality and affection. This instrument, initially designed to evaluate the social well-being of families, comprises 11 items in which participants are asked to rate their level of agreement on a Likert scale ranging from 1, which represents complete agreement or satisfaction, to 5, which represents a lack of agreement or satisfaction. Confidentiality and emotional support were scored on a scale of 5 to 25 points and 6 to 30 points, respectively, with estimated minimum scores of 15 points for confidential and 18 points for emotional support. A score is considered good if the sum of the scores between the two dimensions is greater than 33.

As a part of the qualitative approach, a semi-structured interview was performed. We developed the interview script based on the dimensions of social support. Once the interviews were conducted, they were categorized and coded according with the informative support, advice, material aid and affective support.

Finally, a genealogical map was made to outline the familial structure which provided important details of family members and their relationships. Additionally, an ecological map was created for each participant, categorizing their relationships as “*strong*”, “*strong energy flow in both directions*”, “*tenuous*”, “*stressful*”, “*stressful-energy flow from 1 to 2*”, “*stressful-energy flow from 2 to 1*”, or “*stressful-energy flow in both directions*”.

### Determination of sample size

The sample size calculation was based on a previous study using the calculation software G*Power 3 (University of Dusseldorf) [27] with the t test of independent two-sided sample means raised to the power of 0.95, with an error of alpha = 0.05 and a distribution ratio of 1. The expected sample size for this observational study was 46 subjects.

### Data analysis

Numerical data were analyzed and represented with IBM SPSS Statistics for Windows, Version 20.0 (IBM Co.) The data were entered into an anonymous electronic database by A.A.S. This was followed by a second round of data entry conducted by E.R.A. to verify the accuracy of the data. The statistical computations were carried out by the researcher S.M.P. This involved centrality measures, like the Mean and Median, dispersion, including the Standard Deviation and Variance, and location measures, 25th and 75th Percentiles. To verify that variables met the normality assumption, the Shapiro-Wilk test was performed. The statistical significance level was established at p < 0.05.

After interviews were transcribed and reviewed, qualitative data underwent a coding process in which themes and categories were identified. Firstly, a preliminary reading of the transcripts was conducted to have a general understanding. Following this, different social support providers were identified and coded. The coding was conducted manually by S.M.P. by utilizing the highlighting and annotation. Secondly, we proceeded to input each verbatim into a Microsoft Excel spreadsheet (Microsoft Co.). This was done along with the corresponding coding for each verbatim. It should be taken into consideration that it was often for each verbatim to be assigned multiple codes simultaneously. The process was finally reviewed by an independent reviewer, I.M.P. to ensure that all topics were thoroughly addressed. In the final phase of this study, a genogram and ecomap were created using Canva Inc. software (Canva Pty Ltd.), which was carried out by P.A.P. and E.R.A to create a visual representations of the level of satisfaction experienced by women in relation to their relationships.

## Results

### Description of the sample

A total of 49 women were finally included, with a mean age of 57.80 years-old (SD = 13.25). A total of 83 participants were interviewed, of whom 23 did not agree to participate. Of the remaining n = 60 patients, n = 6 were excluded due to medical diagnoses other than FMS and n = 3 were excluded due to failure to complete the assessment session. Two participants (n=2) were ultimately excluded due to medical reasons or transportation issues.

**Fig. 1:**
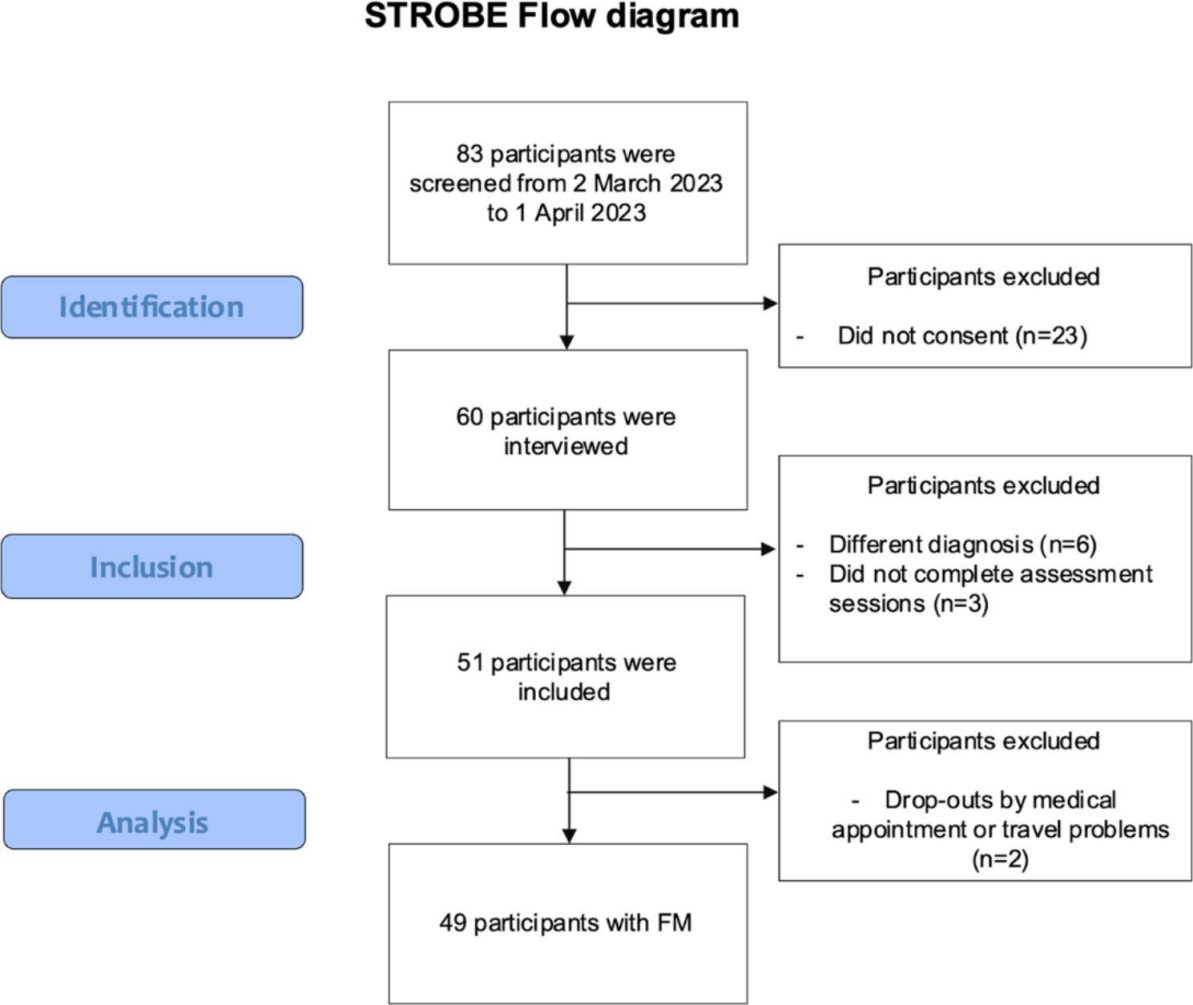
Participants selection process following STROBE criteria

Among all the sample, 40.8% were subjects between the ages of 41 and 50 years-old, while at the opposite, 14.2% of participants aged 20 and 30 years-old. A total of 37 participants (75.6%) in the sample were Spanish, followed by 5 Venezuelans (10.2%), 3 Cubans (6.1%), 2 Germans (4.1%), 1 Italian and 1 Colombian (2.0%) in women with FMS. Most of the sample (59.1%) were married, 24.4% were widows, and only 6 (12.2%) were single. In terms of educational background, most of the sample reached a secondary level (n = 21, 42.8%) while only 6 participants went to university. Currently, most of them were working, although 36.7% (n = 18) of the sample did not. Of those who worked, the type of employment was full time (n = 21, 67.7%) and they mainly lived in sub-urban areas (n = 37, 24.4%).

**Table 1:** Demographic description of the sample

Variable	Frequency	Percentage
**Gender**		
Female	49	100.0%
Male	0	0.0%
**Age**		
**Mean**	57.80 (13.25)	
20–30	7	14.2%
31–40	14	28.6%
41–50	20	40.8%
> 50 years	8	16.3%
**Nationality**		
Spain	37	75,6%
Venezuela	5	10.2%
Cuba	3	6,1%
Germany	2	4.1%
Italy	1	2.0%
Colombia	1	2.0%
**Marital status**		
Widow	12	24.4%
Married	29	59.1%
Single	6	12.2%
Other	2	4.1%
**Educational background**		
Elementary	17	34.7%
Secondary	21	42.8%
Baccalaureate	5	10.2%
University	6	12.2%
**Employment status**		
Employed	12	24.4%
Self-employed	19	38.7%
Unemployed	10	20.4%
Inactive	8	16.32%
**Type of employment contract**		
Part-time	10	32.2%
Full-time	21	67.7%
**Place of residence**		
Sub-urban	37	75.5%
Peri-urban	12	24.4%

### Description of the study variables

Social support measured by MOS-SSS scored 68.6 (SD = 16.3). If we analyze according to different domains of the scale, we can find that the effective support for the low-income FMS patient sample is close to or even lower than the reference values ​​validated for high-income healthy women. Regarding emotional and informational support, the average of patients with FMS was 27.9 (SD = 7.83) compared to the average in the healthy population 33.4 (SD = 6.0) points.

The average value of tangible support obtained was 14.5 (SD = 4.64) points, which means an average data similar to the reference value for healthy women 16.4 (SD = 3.3) points. The dimensions of positive social interactions and affective support have a behavior similar to the previous ones, with an average value somewhat lower than the cut-off value for low-income women with FMS 14.3 (SD = 3.86) and 11.9 (SD = 3.14) respectively.

Concerning the study variables, the mean value of the Perceived Social Support Questionnaire measured by Duke-UNC Functional Social Support (Duke-UNC 11) questionnaire was 38.0 (SD = 9.74), which means that patients perceived support as insufficient and not up to normal levels. Table 2. Description of quantitative variables.

**Table 2:** Description of quantitative variables

					Saphiro-Wilk		Percentiles	
	Mean	Median	SD	Var.	W	p	25th	75th
**Emotional/Informational Support** (MOS-SSS)	27.9	28	7.8	61.3	0.950	0.521	25.50	33.0
**Tangible support** (MOS-SSS)	14.5	16	4.6	21.5	0.931	0.280	11.50	17.5
**Positive social interactions** (MOS-SSS)	14.3	15	3.8	14.9	0.960	0.699	12.00	16.5
**Affective support** (MOS-SSS)	11.9	13	3.1	9.8	0.799	0.004***	11.00	13.5
**MOOS-Total**	68.6	68	16.3	26.9	0.973	0.898	62.50	79.5
**Perceived social support** (Duke-UNC-11)	38.0	38	9.7	94.8	0.946	0.457	30.50	46.5

*Note*: Shapiro-Wilk normality test violation indicated non-normality distribution of sample (*) p < 0.05, (**) p < 0.01, (***) p < 0.001

The qualitative analysis consisted of the evaluation of the social networking to identify the degree of satisfaction experienced with their relationships.

### Informative support

Among the sample, 73.33% of women diagnosed FMS consider their partner as the primary support person with an average score of satisfaction of 8.09 out of 10 (SD = 1.92).

**Quote 1** “[…] *My husband is the person who helps me the most when I am at home and I have a pain crisis. He helps me, for example, make food or hang the clothes* […]” (**Participant 4**).

On the other hand, 10 of them, that is, 66.67% relied on their children, with an average satisfaction score of 7.1 ± 2.23 out of 10.

**Fig. 2:**
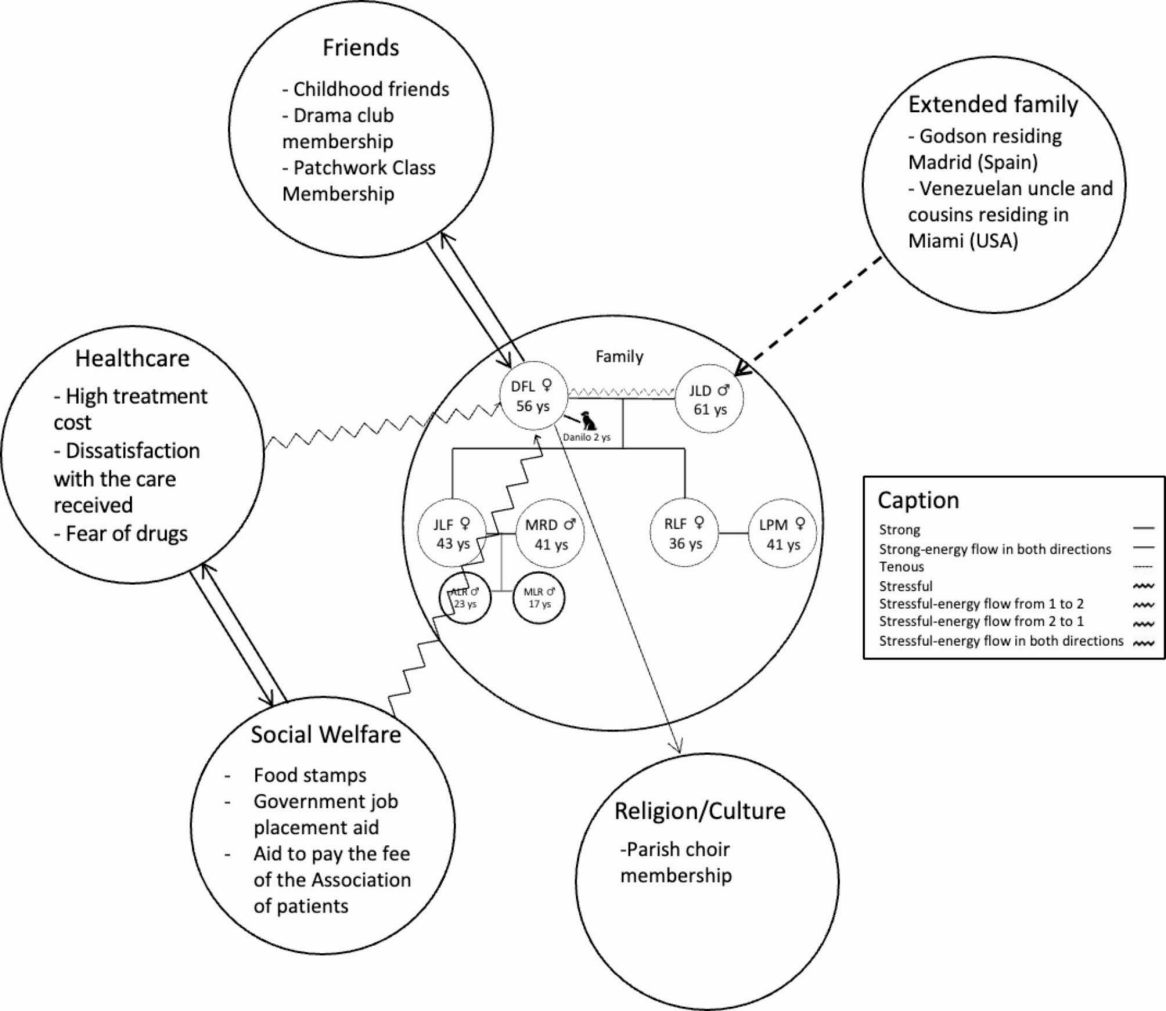
Participant genogram and ecomap of Social Support

**Quote 2** “[…] *I don’t like to talk about the illness, but if I have to, I usually tell my daughter because my husband gets angry when he sees me complaining about pain. He helps me with other things, but I prefer to talk to her about the illness* […]” (**Participant 2**). Figure 2. Genogram and ecomap of Social Support of participant 2.

Parents account for the support of 13.33% of women, with an average score of 10 out of 10 in satisfaction. In addition, 26.66% of them considered their siblings as support, acquiring an average of 9.33 (SD = 1.15) out of 10. The same percentage considered being supported by friends with an average satisfaction of 6.67 (SD = 3.51) over 10. Finally, for this variable only 1 woman, which represented 6.67%, did not rely on anyone and just one relied on “others”, in this case her neighbors, with a satisfaction of 7 out of 10.

### Advice

60% of the participants receive adviced from their partners obtaining an average satisfaction score of 7.44 (SD = 2.92) out of 10.

**Quote 3** “[…] *When I start to have pain that doesn’t let me move, I usually talk to my partner who always gives me advice on how to get on, sit or shower* […]*”* (**Participant 2**).

On the other hand, 46.67% relied on their children, with an average score of 8.43 (SD = 2.37) out of 10 satisfactions. In terms of parental support, no women selected this option. However, 2 of them, which constituted 13.33% of the total consider as support their siblings, being the satisfaction of 10 in both. Friends had also been supportive in 53.33% of women, with a satisfaction of 8.63 (SD = 1.19) out of 10.

**Quote 4***“*[…] *I usually listen to the friends I have in the association because they are the only ones who, having the same illness, are able to understand how I feel when the pain comes* […]*”* (**Participant 11**).

Finally, for this variable 1 woman, that is, 6.67% did not rely on anyone, and another of them, being the same percentage, relied on “others”, in this case their patients association, with a satisfaction of 8 out of 10.

### Material aid

73.33% relied on the couple, obtaining an average of satisfaction of 8.64 (SD = 2.16) out of 10.

**Quote 5**“[…] *My boyfriend is the one who takes me to the doctor or the physiotherapist that I go to once a week* […]” (**Participant 9**).

**Quote 6**“[…] *My husband is the one who usually goes to the bank or shopping at the supermarket when I am in pain. Thank goodness I have it nearby because otherwise I would have to do it myself and it would take me all morning to do it since I’m alone* […]” (**Participant 10**).

On the other hand, 20% material aid relied on their children, with an average satisfaction of 9.0 over 10 (SD = 0.5). With regard to parental support, it constituted 20% of cases, obtaining an average satisfaction score of 10. The same frequency (20%) considered their siblings as support, acquiring an average of 9.33 (SD = 1.15) out of 10 satisfaction. However, friends were not selected as support in any case. Finally, 4 women chose “others” as support, 6.67% of them their cousins with a score of 8 out of 10, and the other 3, which represented 20%, their grandchildren, having an average satisfaction of 10 of 8.33 (SD = 2.89).

### Affective support

Among all the women in the sample, 60% trusted their partners to feel accompanied or received some emotional support, obtaining an average satisfaction score of 7.67 (SD = 2.35) out of 10.

**Quote 7** “[…] *My husband tells me every day I have to go out that I can’t stay home. This weekend we went for a walk on the beach and then we went to eat together, which I hadn’t been able to do for more than 3 months due to the last pain crisis* […]” (**Participant 17**).

On the other hand, 66.67% relied on their children, with an average score of 6.3 (SD = 2.83) out of 10 satisfactions. Parents were a support in 13.33% of women, being 10 the average satisfaction of both. In addition, 26.66% of women considered their brothers a support in this variable, having an average satisfaction of 10 out of 10. Friends had also been supportive in 46.67% of cases, with an average of 7.71 (SD = 1.6) out of 10 satisfactions.

**Quote 8** “[…] *My friends help me a lot and encourage me to leave the house. Sometimes I go with them on hiking trips organized by the Association itself* […]” (**Participant 9**).

Finally, for this variable only one, which constituted 6.67% of the total sample relied on “others”, in this case their pet, with a satisfaction of 10.

## Discussion

The purpose of this study was to analyze social support in low-income women with FMS in the sub-urban and peri-urban area of ​​Tenerife (Canary Islands, Spain). According to our results, FMS women who lived in the suburbs and experienced financial difficulties received significantly less social support than middle-and upper- income women living in affluent areas. This result highlights the main influence of socioeconomic status on social support for women affected by FMS, as reported previously by Londoño et al. (2012) [28].

Providing emotional and informative support obtained the highest ratings among all social support dimensions assessed, followed by tangible support, positive social interactions and affective support.

The lack of informative support compared to healthy remains an unfinished issue for women with FMS, as a large number of patients are unaware of what social and health services are available for them and how this free public assistance can support them in managing their disease. This cannot be explained by their lack of education. Most women of the sample have secondary education, while the rest have completed primary level. The above may be attributed to the lack of specific health education among the population [6].

On the other hand, when patients need information about their condition, they normally asks the family of origin.. These results outline the relevant role of the born family when assessing who they trust to support the disease. In other cases, the procreative family plays a role in coping with the disease. About 75% of women with FMS in our sample reported their partners provide the necessary support for their needs, while 66,67% of their sons also support them in coping with their condition. Our results are consistent with those of Granero-Molina et al. (2018) [10], who concluded that partners provide the most important social support to women with FMS. This emphasizes the importance of having a supportive partner in managing the condition and improving overall well-being.

Nevertheless, marital relationships are deeply influenced by the illness of the couple. In fact, by having to play the role of caregiver and provide social support, partner satisfaction tends to decrease significantly [29] These findings are in line with our previous studies, where we determined that the resistance of husbands to provide care for their wives can often increase the risk of divorce. In contrast, when family members are the main social support providers for those suffering from chronic pain, like FMS patients, Fernández-Peña et al. (2018) [30] consider that the importance of non-family providers and other people within their social networks that do not offer direct help are also essential components of the caring support.

Despite financial worries, FMS women are not concerned for their material needs so tangible support level is in line with those of healthy women. For many of them spiritual support is equally, if not more, important to them. Money is, for the bourgeois, an end, not a means. Instead, for the peasants, money is a means to survive. The rural to urban migration, that is, the abandonment of the countryside to the suburbs in search of better living conditions, a phenomenon in which many recruited participants in this study have been involved, imposes a peasant way of life on the city.

A majority of participants in our sample reported that their partners and friends were the primary providers to fulfilling companionship during leisure activities and even outside of family settings. In our previous publication, we highlighted the importance of associationism since it favors the relationship between equals, which is beneficial to them. This fosters emotional and social support which not only help to fight isolation but also increases the visibility of FMS within society [6].

Compared to those who are in good health, the majority of women reported experiencing a lack of affective support. According to the results of our surveys , patients prioritize emotional support from their partners as the most important form of support. In a second place, friends are also recognized as a key provider of emotional support in nearly half of the sample. Interestingly, a small percentage of participants (around 10%) who are dealing with pain look to their pets for emotional support. Although the data is inconclusive, pets can help improve their owners’ mood.

When assessing the two dimensions of confidentiality and emotional support, it is evident that the level of support is higher in the Canary Islands (mean = 38.00, SD = 9.7), while it is lower in Madrid (Community of Madrid, Spain), Ávila (Castilla-León, Spain) (mean = 35.17,SD 11.34) and Córdoba (Andalusia, Spain) (mean = 29.8,SD = 15.1) [22] These results have several possible explanations.

The character of the Canarian people is the result of a complex interaction of geographical, cultural, historical and social factors, which can contribute to shaping a particular coping style. Unlike the Peninsula, the size of the territory is small. The territory of Tenerife island is small, it is approximately 2,034 km², where the highest town is located at 1400 m. The towns and villages are close, where getting to know each other is common among all of them. It works like an extended family in which even the neighbors are part of it.

Secondly, it is known from the literature that Canary Islanders in the 20th century primarily directed their emigration to Cuba and Venezuela. These are the main sources of return migrations of descendants of canary immigrants currently recorded in the archipielago. These descendants preferably settle in the sub-urban and periurban belts of cities like Santa Cruz de Tenerife and San Cristóbal de La Laguna. They coexist with the local population in a diverse and multicultural environment in which mutual respect and cultural interaction promotes social cohesion. This is achieved through the establishment of horizontal relationships based on equality, rather than vertical ones. As a result, these factors promote confidentiality and provide emotional support for those women complaining of FMS.

### Limitations

In this research, there are several limitations that can affect the results of the study. First, the lack of previous research on our topic was a major limitation of our work, resulting in a lack of reliable or accessible data.

Secondly, the patients that were diagnosed with FMS in our study were not a uniform group. Within this group, there were women who had been diagnosed with FMS and CFS, which produces multiple symptoms in each individual.

Likewise, due to differences in age and the sociocultural context in which the disease occurs, there may be differences in the interpretation of the disease and perceptions of social support. Therefore, it is inappropriate to extrapolate to other cultures.

On the other hand, one of the limitations found has to do with the sample size. Despite our best efforts, we have not been able to make it large enough to ensure fair representation. In this sense, it is important to consider that our study was carried out exclusively on poor women with FMS. Hence, it would not be appropriate to generalize our findings to the entire popultation.

## Conclusion

The socioeconomic status of low-income women with FMS living in sub-urban and peri-urban areas of Tenerife (Canary Islands, Spain) influences on their social support, with affective support and confidentiality being the most affected dimensions. The lack of targeted health education could partially account for their unawareness of available support resources. Both tangible and emotional support are equally necessary for them. Coping with the disease is heavily influenced by the family, especially the partner. Multicultural environments such as suburbs provide a platform for developing horizontal relationships, which aid in combating FMS.

## Data Availability

Please contact the authors for data requests.

## References

[1] Berwick R, Barker C, Goebel A (2022) The diagnosis of fibromyalgia syndrome. Clin Med (Lond) 22(6). 10.7861/clinmed.2022-0402 10.7861/clinmed.2022-0402 PMC9761415 36427885

[2] Sarzi-Puttini P, Giorgi V, Marotto D, Atzeni F (2020) Fibromyalgia: an update on clinical characteristics, aetiopathogenesis and treatment. Nat Rev Rheumatol 16(11). 10.1038/s41584-020-00506-w 10.1038/s41584-020-00506-w 33024295

[3] Altomonte L, Atzeni F, Leardini G et al (2011) Fibromyalgia syndrome: preventive, social and economic aspects. Reumatismo 60(1s). 10.4081/reumatismo.2008.1s.70 10.4081/reumatismo.2008.1s.70 18852910

[4] Crooks VA (2007) Exploring the altered daily geographies and lifeworlds of women living with fibromyalgia syndrome: a mixed-method approach. Soc Sci Med 64(3). 10.1016/j.socscimed.2006.09.025 10.1016/j.socscimed.2006.09.025 17079063

[5] Biccheri E, Roussiau N, Mambet-Doué C, Fibromyalgia (2016) Spirituality, coping and quality of life. J Relig Health 55(4). 10.1007/s10943-016-0216-9 10.1007/s10943-016-0216-9 26922751

[6] Martín Pérez IM, Martín Pérez SE, Martínez Rampérrez R, Vaswani Vaswani SS, Dorta Borges MF (2023). Conocimientos, actitudes y creencias hacia la enfermedad en mujeres con fibromialgia. Un Estudio Cualitativo basado en un grupo focal. Rev Soc Esp Dolor.

[7] Che X, Cash R, Ng SK, Fitzgerald P, Fitzgibbon BM (2018) A systematic review of the processes underlying the Main and the Buffering Effect of Social Support on the experience of Pain. Clin J Pain 34(11). 10.1097/AJP.0000000000000624 10.1097/AJP.0000000000000624 29697476

[8] Guariglia P, Palmiero M, Giannini AM, Piccardi L (2023) The Key Role of Empathy in the relationship between Age and Social Support. Healthc (Switzerland) 11(17). 10.3390/healthcare11172464 10.3390/healthcare11172464 PMC10487866 37685497

[9] Bolwijn PH, Van Santen-Hoeufft MHS, Baars HMJ, Kaplan CD, Van Der Linden S (1996) The social network characteristics of fibromyalgia patients compared with healthy controls. Arthritis Rheum 9(1). 10.1002/art.1790090106 10.1002/art.1790090106 8945109

[10] Granero-Molina J, Matarín Jiménez TM, Ramos Rodríguez C, Hernández-Padilla JM, Castro-Sánchez AM, Fernández-Sola C (2018) Social support for female sexual dysfunction in Fibromyalgia. Clin Nurs Res 27(3). 10.1177/1054773816676941 10.1177/1054773816676941 29421939

[11] Gündüz N, Üşen A, Aydin Atar E (2019) The impact of perceived social support on anxiety, depression and severity of pain and burnout among Turkish females with fibromyalgia. Arch Rheumatol 34(2). 10.5606/ArchRheumatol.2019.7018 10.5606/ArchRheumatol.2019.7018 PMC6719574 31497765

[12] Lynch-Jordan AM, Sil S, Bromberg M, Ting TV, Kashikar-Zuck S (2015) Cross-sectional study of young adults diagnosed with Juvenile Fibromyalgia: Social Support and its impact on Functioning and Mood. J Adolesc Health 57(5). 10.1016/j.jadohealth.2015.07.015 10.1016/j.jadohealth.2015.07.015 26372369

[13] Ruschak I, Toussaint L, Rosselló L, Martín CA, Fernández-Sáez J, Montesó-Curto P (2022) Symptomatology of Fibromyalgia Syndrome in men: a mixed-method pilot study. Int J Environ Res Public Health 19(3). 10.3390/ijerph19031724 10.3390/ijerph19031724 PMC8834813 35162747

[14] Chokshi DA (2018) Income, poverty, and health inequality. JAMA 319(13). 10.1001/jama.2018.2521 10.1001/jama.2018.2521 29614168

[15] Franks HM, Cronan TA, Oliver K (2004) Social support in women with fibromyalgia: is quality more important than quantity? J Community Psychol 32(4). 10.1002/jcop.20011

[16] Kengen Traska T, Rutledge DN, Mouttapa M, Weiss J, Aquino J (2012) Strategies used for managing symptoms by women with fibromyalgia. J Clin Nurs 21(5–6). 10.1111/j.1365-2702.2010.03501.x 10.1111/j.1365-2702.2010.03501.x 21323780

[17] Cooper S, Gilbert L (2017) An exploratory study of the experience of fibromyalgia diagnosis in South Africa. Health (United Kingdom) 21(3). 10.1177/1363459316677623 10.1177/1363459316677623 28521648

[18] Reig-Garcia G, Bosch-Farré C, Suñer-Soler R et al (2021) The impact of a peer social support network from the perspective of women with fibromyalgia: a qualitative study. Int J Environ Res Public Health 18(23). 10.3390/ijerph182312801 10.3390/ijerph182312801 PMC8657284 34886527

[19] Schoofs N, Bambini D, Ronning P, Bielak E, Woehl J (2004) Death of a lifestyle: the effects of social support and healthcare support on the quality of life of persons with fibromyalgia and/or Chronic Fatigue Syndrome. Orthop Nurs 23(6). 10.1097/00006416-200411000-00005 10.1097/00006416-200411000-00005 15682879

[20] West C, Buettner P, Stewart L, Foster K, Usher K (2012) Resilience in families with a member with chronic pain: a mixed methods study. J Clin Nurs 21(23–24). 10.1111/j.1365-2702.2012.04271.x 10.1111/j.1365-2702.2012.04271.x 23020890

[21] Fernández-Peña R, Molina JL, Valero O (2018) Personal network analysis in the study of social support: the case of chronic pain. Int J Environ Res Public Health 15(12). 10.3390/ijerph15122695 10.3390/ijerph15122695 PMC6313565 30501074

[22] Moral RR, Alamo MM, De Torres LP, Aguayo Galeote MA (1997) Biopsychosocial features of patients with widespread chronic musculoskeletal pain in family medicine clinics. Fam Pract 14(3). 10.1093/fampra/14.3.242 10.1093/fampra/14.3.242 9201500

[23] Guerro-Prado D, Echeverria N, Jiménez L et al (2011) Quality of life and social adaptation in women suffering from fibromyalgia. Eur Psychiatry 26(S2). 10.1016/s0924-9338(11)73362-4

[24] Knottnerus JA, Tugwell P (2010) Real world research. J Clin Epidemiol 63(10). 10.1016/j.jclinepi.2010.08.001 10.1016/j.jclinepi.2010.08.001 20728043

[25] Merino-Soto C, Núñez Benítez MÁ, Domínguez-Guedea MT et al (2023) Medical outcomes study social support survey (MOS-SSS) in patients with chronic Disease: a psychometric assessment. Front Psychiatry 13. 10.3389/fpsyt.2022.1028342 10.3389/fpsyt.2022.1028342 PMC9874003 36713918

[26] Bellon Saameno Ja, Delgado Sa, del Castillo L, Lardelli JD (1996) CP. Validez y fiabilidad del cuestionario de apoyo social funcional Duke-UNC-11. Aten Primaria; 18 8962994

[27] Faul F, Erdfelder E, Lang AG, Buchner A (2007) G*Power 3: a flexible statistical power analysis program for the social, behavioral, and biomedical sciences. Behav Res Methods 39. 10.3758/BF03193146 10.3758/bf03193146 17695343

[28] Arredondo NHL, Rorgers HL, Tang JFC (2012). Validación en Colombia del cuestionario MOS de apoyo social. Int J Psychol Res (Medellin).

[29] Steiner JL, Bigatti SM, Hernandez AM, Lydon-Lam JR, Johnston EL (2010) Social Support mediates the relations between role strains and marital satisfaction in husbands of patients with Fibromyalgia Syndrome. Fam Syst Health 28(3). 10.1037/a0020340 10.1037/a0020340 20939626

[30] Fernández-Peña R, Molina JL, Valero O (2020) Satisfaction with social support received from social relationships in cases of chronic pain: the influence of personal network characteristics in terms of structure, composition and functional content. Int J Environ Res Public Health 17(8). 10.3390/ijerph17082706 10.3390/ijerph17082706 PMC7215382 32326411

